# SUNPLIN: Simulation with Uncertainty for Phylogenetic Investigations

**DOI:** 10.1186/1471-2105-14-324

**Published:** 2013-11-15

**Authors:** Wellington S Martins, Welton C Carmo, Humberto J Longo, Thierson C Rosa, Thiago F Rangel

**Affiliations:** 1 Institute of Informatics, Federal University of Goiás, Goiânia, Brazil; 2 Department of Ecology, Federal University of Goiás, Goiânia, Brazil

## Abstract

**Background:**

Phylogenetic comparative analyses usually rely on a single consensus phylogenetic tree in order to study evolutionary processes. However, most phylogenetic trees are incomplete with regard to species sampling, which may critically compromise analyses. Some approaches have been proposed to integrate non-molecular phylogenetic information into incomplete molecular phylogenies. An expanded tree approach consists of adding missing species to random locations within their clade. The information contained in the topology of the resulting expanded trees can be captured by the pairwise phylogenetic distance between species and stored in a matrix for further statistical analysis. Thus, the random expansion and processing of multiple phylogenetic trees can be used to estimate the phylogenetic uncertainty through a simulation procedure. Because of the computational burden required, unless this procedure is efficiently implemented, the analyses are of limited applicability.

**Results:**

In this paper, we present efficient algorithms and implementations for randomly expanding and processing phylogenetic trees so that simulations involved in comparative phylogenetic analysis with uncertainty can be conducted in a reasonable time. We propose algorithms for both randomly expanding trees and calculating distance matrices. We made available the source code, which was written in the C++ language. The code may be used as a standalone program or as a shared object in the R system. The software can also be used as a web service through the link: http://purl.oclc.org/NET/sunplin/.

**Conclusion:**

We compare our implementations to similar solutions and show that significant performance gains can be obtained. Our results open up the possibility of accounting for phylogenetic uncertainty in evolutionary and ecological analyses of large datasets.

## Background

Phylogenetic trees are hypothetical statements about the evolutionary relationship among species. The methods to generate the most probable evolutionary hypothesis are usually based on search algorithms that try to maximize the fit between tree topology and the data, given a model of evolution [1]. Although biologists increasingly aim to take phylogeny into account in their studies (e.g. [2]), phylogenetic uncertainty is routinely ignored. An important source of phylogenetic uncertainty arises when biologists use incomplete phylogenetic data to make inferences about evolutionary mechanisms that supposedly affect a group of species [3,4]. In particular, phylogenetic uncertainty may arise from three distinct sources: (1) weak empirical support for hypothesized relationships among species in a given clade, (2) errors associated with tree topology and branch lengths, and (3) incomplete and unrepresentative sampling of known species. Biologists have employed different strategies to deal with phylogenetic uncertainty. The first approach is to focus only on clades for which complete phylogenies are available, but this strategy restricts studies to a very small number of groups and can undermine assemblage-level studies if only certain species can be included. A second and more radical strategy is to ignore the species that are absent from the available phylogeny, therefore assuming that evolutionary processes captured by sampled species can be extrapolated to the missing species [5]. Finally, the most common approach consists in assembling supertrees from small overlapping trees, assigning unknown evolutionary relationships among species as polytomies (nodes with more than two derived branches). Clearly, these strategies are sub-optimal, as they hide or ignore phylogenetic uncertainty.

Neglecting phylogenetic uncertainty may seriously affect inference of evolutionary processes that drive biological patterns. Incomplete phylogenies composed of biased samples of species can potentially provide empirical evidence towards an incorrect evolutionary model, therefore misleading biological inference. For example, one of the goals of Conservation Biology is to design a network of conservation units that maximize the protection of biodiversity. However, given a biased and incomplete phylogeny the estimate of biodiversity across regions may support the creation of a biological reserve in a locality that harbors sub-maximum phylogenetic diversity.

Even in the total absence of genomic information about a species, morphological, behavioral and ecological characters can provide important clues about the evolutionary relationship with other species [6]. In general, these non-genetic sources of information are not accurate, as they are insufficient to indicate the closest species or phylogenetic distances between species. However, such additional sources of information can be reliably used in evolutionary studies provided that the statistical uncertainty can be properly estimated [3,6]. One of the most accepted strategies to account for uncertainty in statistical analysis is Monte Carlo simulation, which rely on repeated random sampling to model phenomena with substantial uncertainty in the input data [5,6].

The advantage of estimating statistical error associated with inference based on uncertain data comes at the cost of highly replicated computations. The computational demands required to replicate statistical analyses that rely on phylogenetic information have prevented biologists to fully employ simulation methods in large-scale ecological and evolutionary studies. Few notable exceptions are available in the literature. For example, [7] used simulation of incomplete phylogenetic information to estimate rates of diversification of fishes, and [8] developed likelihood approaches to infer the effect of trait on diversification rates, testing their new method using simulated phylogenies with varying degree of resolution. Finally, [9] employed the method described in this paper to study the consequences of extinction of endangered frogs to the biodiversity, indicating regions across the New World where biodiversity is significantly threatened. The tools developed in this study have the potential to enable biologists to expand the scale of biodiversity analysis while accounting for uncertainty in the available phylogenetic information. Potential applications of this method are unlimited, ranging for the study of evolution of species traits, distribution of biodiversity in time and space, and designing network of reserves to protect biodiversity.

To account for phylogenetic uncertainty in statistical analyses using simulation, one would have to build a large set of phylogenetic trees, in which *Phylogenetic Uncertain Taxa* (PUT) would be randomly assigned to partially known trees (e.g. built from molecular data). However, as some phylogenetic knowledge about PUT is usually available, the insertion point for each PUT should not be totally random across the phylogeny. Hence, for each PUT one must determine the *Most Derived Consensus Clade* (MDCC) that unequivocally contains the PUT. This is done using all available biological information and, when necessary, classification based on the best available taxonomy. Thus, a MDCC defines the sub-tree that is known to include the species, and constrains the scope of random allocation of the PUT. This repeated insertion of PUTs in the partially known tree produces expanded trees that can be used in statistical analyses required for the simulation process.

The information contained in the topology of the phylogenetic trees is of maximum interest to biologists, as inferences of evolutionary processes are derived from the phylogenetic relationship between species. This relationship can be captured by the pairwise phylogenetic distance between species and, when multiple species are included in the analysis, such information can be conveniently stored in a squared distance matrix [5], the so called *Patristic Distance Matrix* (PDM). A patristic distance is the sum of the lengths of the branches that link two species in a tree. Some of the statistical methods used to study evolutionary processes require the raw or standardized patristic distance matrix (e.g. estimate phylogenetic diversity), whereas other methods require the transformation of the distance matrix into a variance-covariance matrix (e.g. linear regression analysis). In any case, if there is uncertainty in the relationship among species, simulations can be used to estimate the phylogenetic uncertainty by generating multiple random phylogenetic trees with phylogenetic uncertain species inserted into the main tree. Thus, in addition to the computational burden of inserting PUT into a tree, one must compute a pairwise distance matrix for each randomly expanded phylogenetic tree. Of course, the next step in the phylogenetic comparative analysis consists in the replication of the statistical methods using each phylogenetic distance matrix. The averaged phylogenetic measure across replicates of the analysis captures the best estimate of the true parameter (e.g. phylogenetic diversity of species), whereas the variance of the phylogenetic measure combines the error due to sampling size (number of species) and phylogenetic uncertainty in the statistical analysis.

There have been some proposals to integrate non-molecular phylogenetic information into incomplete molecular phylogenies in order to conduct phylogenetic comparative analyses. Two main approaches have been proposed for the analyses of diversification. A skeletal tree approach [8], that works by collapsing the under sampled clades and producing a complete but terminally unresolved tree, and an expanded tree approach that creates a complete and terminally resolved tree by randomly inserting missing taxa along the branches belonging to its likely clade. The former approach was implemented in the diversitree R package [10], whereas the latter approach was implemented in [7] as an R script using APE functions [11]. In this work we use the expanded tree approach since many statistical methods used to study evolutionary processes require a complete and terminally resolved tree. Our approach involves an algorithm for randomly expanding trees that performs a single tree traversal for each tree being expanded.

As for the calculation of patristic distance matrices, there are many software tools that provide this functionality, e.g. [11-13]. However, most implementations were not meant to support simulation studies and thus are not prepared to deal efficiently with a large number of trees. To calculate each matrix element, i.e. the pairwise phylogenetic distance between species, an efficient method has to be used to sum up all branch lengths connecting them. Otherwise, too much time is spent recalculating distances along the branches. This situation is aggravated in a scenario containing a large number of trees. We use a heavy chain decomposition (see Subsection Distance computation) to structure the expanded trees and thus produce a fast solution to the pairwise distance matrix calculation.

The work presented in this paper aims at contributing to conduct large-scale phylogenetic comparative simulation that take uncertainty into account. We present efficient algorithms and implementations to generate random expanded trees, with the insertion of phylogenetic uncertain taxa (PUT), and to calculate patristic distance matrices, both commonly used in large-scale statistical analyses. The rest of the paper is organized as follows. In the next section we present the algorithms proposed. The experimental results are shown in the following section, together with a comparative analysis with respect to well-known tools. Finally in the last section we present the conclusions and future works.

## Implementation

In this paper we present a computational tool named SUNPLIN (Simulation with Uncertainty for Phylogenetic Investigations) that allows one to use an existing phylogenetic tree, along with a list of species to be inserted, and to produce randomly expanded versions of the input tree as well as distance matrices for the corresponding trees. The input phylogenetic tree is assumed to be in the Newick format [14], one of the most widely used tree formats, while the species to be inserted should be in a plain file containing one species per line. The most derived consensus clade (MDCC) must be indicated following the species name, separated by a space.

In this section we use the terminology given by the following definitions: 

• Species and ancestors in a phylogenetic tree are both named *nodes*;

• The *descendants* of a node *u* are all nodes in the subtree rooted at *u* but not including *u*;

• The term *tree* is used as abbreviation of the term *phylogenetic tree*;

• Nodes without descendants (species) in the tree are referred to as *leaf nodes*;

• Ancestor nodes are named *internal nodes*;

• The internal node without ancestors is the *root node* of the tree;

• The direct ancestor of a node *u* is referred to as the *parent node* of *u*;

• If a node *p* is an ancestor node, each of its direct descendants is a *child node* of *p*;

• A line connecting a parent node *p* to one of its child node *c* is a *branch* or an *edge*;

• Each branch has a *length* which is a real number;

• A node *u* and all its descendants form a *subtree* with *u* as root node;

• A *path* in the tree from node *u* to a node $u^{\prime }$ is the sequence 〈*v*_0_,*v*_1_,…,*v*_*t*_〉 of nodes such that, *u* = *v*_0_, $u^{\prime }=v_{t}$ and there is a branch connecting *v*_*i*_ to *v*_*i* + 1_, for 0≤*i*≤*t*-1.

**Figure 1:**
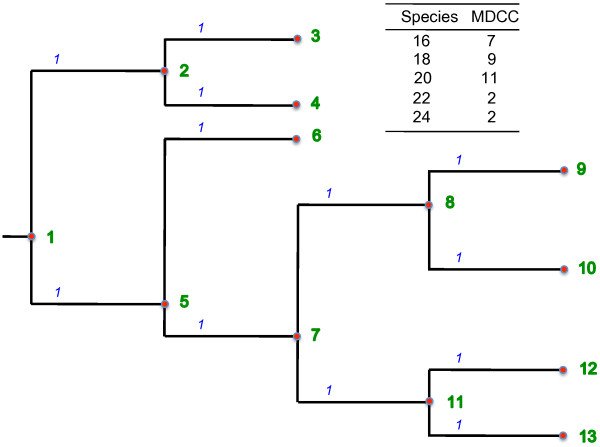
**(Left) Phylogenetic tree and (top right) the species to be inserted.** Input data representation. Phylogenetic tree and the species to be inserted.

Figure 1 illustrates an example tree with numbered nodes. The root of the tree is node 1. Nodes 3, 4, 6, 9, 10, 12 and 13 are leaf nodes. For the sake of simplification, the tree is made binary and all branch lengths are assigned the value 1.

In the following subsections, we present the tree expansion problem and the distance computation problem. This follows a detailed description of the proposed algorithms, auxiliary functions, examples and a complexity analysis.

### Tree expansion

The tree expansion problem has as input the following data: 

a) A phylogenetic tree *T*_0_;

b) An integer number *m*;

c) A set $S=\{\langle s_{1},v_{\text{mdcc}}^{1}\rangle ,\ldots ,\langle s_{\left|S\right|},v_{\text{mdcc}}^{\left|S\right|}\rangle \}$, where each pair $\langle s_{i},v_{\text{mdcc}}^{i}\rangle$ in *S* is composed by a species *s*_*i*_ to be inserted in the subtree with root $v_{\text{mdcc}}^{i}$ in copies of *T*_0_.

The tree expansion problem consists in making *m* copies of the original tree *T*_0_ and inserting all the species of *S* in each of the copied trees. The insertion of a species *s*_*i*_ from *S* must occur in a place randomly chosen in the subtree with root in node $v_{\text{mdcc}}^{i}$. Thus, a solution to the problem produces *m* expanded trees *T*_1_,…,*T*_*m*_. Each one of the expanded trees contains the same nodes (nodes in the original tree and nodes corresponding to the inserted species), however they are different from each other, because each species *s*_*i*_ in *S* was inserted randomly in distinct positions in the subtree with root $v_{\text{mdcc}}^{i}$ in each tree *T*_*j*_, 1≤*j*≤*m*.

In this work, we consider two possible random insertion methods: the *node-based* method and the *branch-based* method. The first method chooses randomly a node *p* in the subtree with root in the MDCC and inserts the species as a child of *p*. In this case, the chance of receiving a new species is equal for all nodes. For example, consider a copy *T*_1_ of the tree in Figure 1. The insertion of species 16 in MDCC 7 involves choosing randomly one node among the nodes 7, 8, 9, 10, 11, 12 or 13 as the insertion point.

The branch-based method is characterized by giving to long branches a higher chance of being split due to the insertion of a new species. This is obtained by randomly choosing a number between zero and the sum of branch lengths below the MDCC. This number corresponds to the accumulated lengths of a sequence of branches ordered according to a depth-first traversal in the subtree with the MDCC as root. For example, let us consider the insertion of species 16. The sum of the lengths of branches in the subtree with root in node 7 (the MDCC) equals to 6. We choose randomly a real number between 0 and 6; let us say 4.2. A depth-first walk in subtree with 7 as root reaches the branches in the following order: (7,8), (8,9), (8,10), (7,11), (11,12) and (11,13). We follow the branches in that order accumulating their lengths while this accumulated value is less than or equal to 4.2. Thus, this procedure stops when we reach branch (11,12). Consequently, node 12 is chosen as the insertion point.

Efficient implementation of both insertion methods is crucial to solve the tree expansion problem. An obvious but inefficient solution is to perform a traversal in the subtree with root in the MDCC. During this traversal, nodes are collected, in the case of the node-based method, or branches are collected, in the case of the branch-based method, and the insertion point is chosen randomly in each case. This is inefficient because we have to perform a walk for every subtree and every species to be inserted. Also, the MDCC for some species to be inserted might be located too high in the tree, next to the root, which would imply that almost the whole tree would have to be traversed.

In this article, we propose a method that allows for deciding the insertion point, in both node-based and branch-based methods, with only one depth-first traversal of the original tree *T*_0_. This traversal occurs in a preprocessing phase of the algorithm and computes for each node in the tree some information used to define the insertion point. Our insertion algorithm relies on a numbering of the nodes of *T*_0_ with numbers assigned in an increasing order, starting with number 1 that is assigned to the root of *T*_0_. The numbers are assigned to the nodes through a depth-first search (DFS), following a post-order traversal. In Figure 1, the number of each node is shown just at the right of the node.

The preprocessing phase is performed by the function PreProcessExpansion(). During this phase, the following information is computed for each node *u* of the tree *T*_0_: 

• The number of descendant nodes of node *u*. This counting is represented by the array *descendants*[*u*] in the function.

• The sum of the lengths of branches on the DFS traversal of the tree starting at the root and ending at node *u*. This value is represented by the variable *sumLengths*[*u*] in the function.

#### **Function**PreProcessExpansion(*u*)

For example, consider the tree shown in Figure 1. The number of descendants of node 2 (*descendants*[2]) equals to two, while *descendants*[5] equals to seven. The following branches are reached through a DFS traversal starting at the root node and ending at node 7: (1,2), (2,3), (2,4), (1,5), (5,6), (5,7). The sum of the lengths of these branches (*sumLengths*[7]) equals to six. Analogously, *sumLengths*[9] equals to eight.

The tree expansion method is described in algorithm TreeExpansion. The algorithm receives as input the phylogenetic tree *T*_0_, the number *m* of expanded trees, the set *S* of species to be inserted and the method of insertion to be used.

#### **Algorithm**TreeExpansion(*T*_0_,*m*,*S*,*method*)

**Figure 2:**
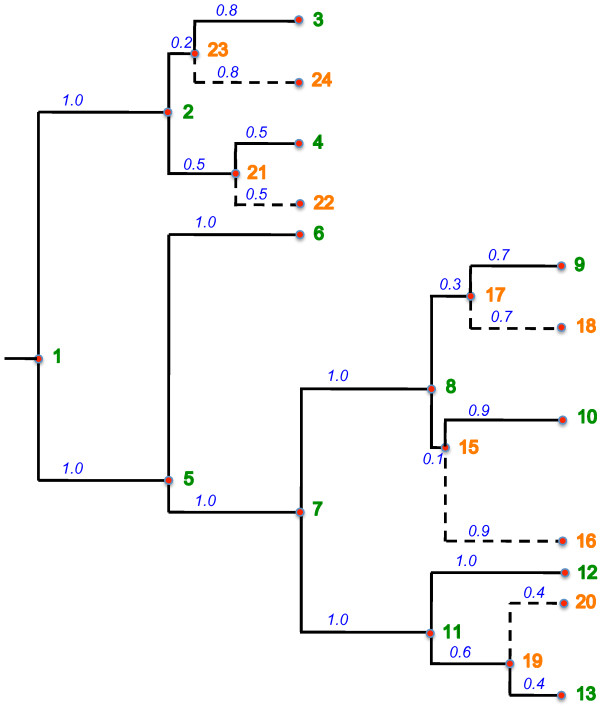
**A possible expanded tree.** A possible final expanded tree where branches connecting inserted species are shown by dashed lines.

Figure 2 shows how the tree in Figure 1 could be expanded by the insertion of the species 16, 18, 20, 22 and 24 using 7, 9, 11, 2 and 2 as the respective MDCCs, that is, *S*={〈16,7〉,〈18,9〉,〈20,11〉,〈22,2〉,〈24,2〉}.

The TreeExpansion algorithm starts by activating the function PreProcessExpansion() to compute the values of variables *descendants*[*u*] and *sumLengths*[*u*] for each node *u* in *T*_0_. Next, at each iteration *i*≤*m*, the algorithm makes a copy *T*_*i*_ of *T*_0_ and for each pair 〈*s*,*v*_*mdcc*_〉 of *S* it chooses randomly an insertion point *p* in the subtree with root in *v*_*mdcc*_ to insert the species *s*. This choosing is done using only information of the original tree *T*_0_, computed during the preprocessing phase. The preprocessing phase takes time *O*(|*T*_0_|) to execute, because it has to traverse through all nodes and branches of the tree *T*_0_ to compute the number of descendants and the sum of branch lengths for each node.

If the insertion method is node-based, a random node must be chosen among all nodes in the subtree with root in node *v*_*mdcc*_. However, because of the numbering strategy used to number nodes in the tree and the previous computation of the number of descendants of each node, the random insertion point can be easily and efficiently computed as shown in line seven of the algorithm. For instance, to insert species 16 in MDCC 7, the algorithm chooses randomly a number in the interval [7, *descendants*[7] + 7], that is, [7, 13]. This interval comprises all node numbers in the subtree with root in node 7 (See Figure 1). The random choosing of the insertion point takes *O*(1), however it is repeated for each of the |*S*| species to be inserted. Thus, the total time spent by the node-based method to insert all the |*S*| species is *O*(|*T*_0_|+|*S*|).

If the insertion method is branch-based, the sum *σ* of lengths of branches in the subtree with root in *v*_*mdcc*_ can be easily computed without traversing the tree. It is given by the computation in line 9 of Algorithm TreeExpansion. For example, the sum of branch lengths in the subtree with root in node 7 is given by *sumLengths*[*descendants*[7] + 7] - *sumLengths*[ 7] = *sumLengths*[6+7]- *sumLengths*[7]=12-6=6. In line 10 of the algorithm, a random value between 0 and the sum *σ* is obtained. In line 11 of the algorithm, the random value obtained is added to the value of *sumLengths*[ *v*_*mdcc*_] and assigned to *δ*. The point of insertion is the node *p* such that *sumLengths*[ *p*] is most similar to *δ*. The algorithm has to search for such *p* among the subtree rooted at *v*_*mdcc*_ that is, it has to search in the interval [*sumLengths*[ *v*_*mdcc*_],*sumLengths*[ *v*_*mdcc*_ + *descendants*[ *v*_*mdcc*_] ]. Once the insertion point *p* has been found, the algorithm TreeExpansion inserts (see line 13) species *s* at node *p* of the copied tree *T*_*i*_, taking time *O*(1) independently of the insertion method used.

The good news is that values in vector *sumLengths*[] are ordered, thus a binary search can be used to find such node *p*, in time $O(\log(\text{descendants}[\;v_{\text{mdc}}]\;))$. The worst case occurs when the MDDC for a species is the root of the tree. In this case, the binary search has to be applied in the whole vector that has size |*T*_0_|. This operation takes time $O(\log|T_{0}|)$. Since the binary search has to be applied for each of the |*S*| species, in the worst case the branch-based mode has execution time $O(|T_{0}|+|S|\;\cdot \log|T_{0}|)$.

### Distance computation

In this section we present the more generic problem of computing distances between nodes in a phylogenetic tree - *node-to-node distance problem*. The case of computing distances between only leaf nodes (patristic distance) can be seen as a subproblem of node-to-node distance problem in which we discard the computation for nodes that are not leaves. We show an efficient solution to the node-to-node distance problem that can be easily adapted to compute the patristic distance, as is explained at the end of this section.

The node-to-node distance problem consists in computing for each pair (*u*,*v*), where *u* and *v* are nodes of a phylogenetic tree *T*, the *distance* between *u* and *v* in *T*. The distance between *u* and *v* corresponds to the sum of branches lengths in the path that connects *u* to *v* in *T*. For example, in Figure 1, the distance between nodes 6 and 13 is the accumulated lengths of branches connecting both nodes, which equals to four.

The objective of the problem is to compute a distance matrix *Dist* of dimension |*T*|×|*T*|, where |*T*| is the number of nodes in *T*. This matrix is symmetric, with zeros in the main diagonal, so we only need to calculate half of the elements of the matrix. However, to calculate a single distance between a pair of nodes we need to sum up the lengths of all branches connecting the nodes. In the worst case, we have a tree whose internal nodes form a list and each internal node has a single leaf node, except the last one which has two leaves (Monophyly). In this case, if one of the elements of a pair is the deepest leaf node and the other is the root of the tree, the computation of the distance of nodes in the pair takes time *O*(|*T*|) to complete. However, the computation of the distance between two nodes is repeated (|*T*|^2^-|*T*|)/2 times, thus the time complexity of the distance matrix calculation becomes *O*(|*T*|^3^).

We propose an algorithm that avoids traversing entire paths repeatedly and thus reduces this complexity to $O(|T{|}^{2}\;\cdot \log|T|)$. Our solution first calculates the distance of every node to the root of the tree. This is done in a preprocessing phase executed by function PreProcessDistance(). The array *distRoot*[] keeps the distance between each node *u* and the root of the tree. This function also computes for each node *u* in the tree the number of descendants of *u*. This information is kept in array *descendants*[].

#### **Function**PreProcessDistance(*u*,*distRoot*)

Once the distances between the root and the other nodes in the tree has been computed, the distance between any two nodes *u* and *v* can be computed by first finding the *Lowest Common Ancestor*(LCA) of *u* and *v*, i.e., the deepest node in the tree that has both nodes as descendants. The distance between the pair of species is then calculated by summing the distances of each node to the root minus twice the distance of the LCA to the root, i.e., *Dist*[ *u*,*v*] = *distRoot*[ *u*] + *distRoot*[ *v*]-2 ·*distRoot*[ *LCA*(*u*,*v*)]. For example, let us consider Figure 1 again. The LCA for nodes 6 and 13 is node 5. The *Dist*[6,13] can be computed as *distRoot*[6] + *distRoot*[13]-2 ·*distRoot*[5], which is equal to 2+4-2. Thus, *Dist*[6,13] = 4.

Since the LCA calculation has to be repeated for each pair of species, this computation has to be performed efficiently. We propose a method to find the LCA of any two nodes in the tree that performs a decomposition of the tree into chains of nodes. Our method is based on the *Heavy Light Decomposition*, a method that was introduced by Sleator and Tarjan [15]. Our solution is named *Heavy Chain Decomposition*.

To explain the heavy chain decomposition we first need to present some definitions. A *chain* from node *u* to one of its descendant node $u^{\prime }$ is the sequence 〈*v*_0_,*v*_1_,…,*v*_*t*_〉 of nodes such that, *u* = *v*_0_, $u^{\prime }=v_{t}$ and *v*_*i*_ is the parent node of *v*_*i* + 1_, for 0≤*i*≤*t*-1. There is a chain from each node *u* to itself, because in this case the chain is the unitary sequence 〈*u*〉. We refer to node *u* as the *leader node* of the chain from *u* to any of its descendant or to itself, because it is the first node of the chain. We define *the cost* of a chain *C* = 〈*v*_0_,*v*_1_,…,*v*_*t*_〉 as:

(1) $$\text{Cost}(C)=\underset{v\in C}{\sum }\text{descendants}[v]$$

That is, *Cost*(*C*) is the accumulated number of descendants of nodes belonging to chain *C*. We define the *heavy chain* for a node *u* as the chain *C* that contains *u* as its leader and conforms to the following conditions: 

• The last node in *C* must be a leaf node;

• *C* is the chain with the highest cost among every chain from *u* to leaf nodes.

**Figure 3:**
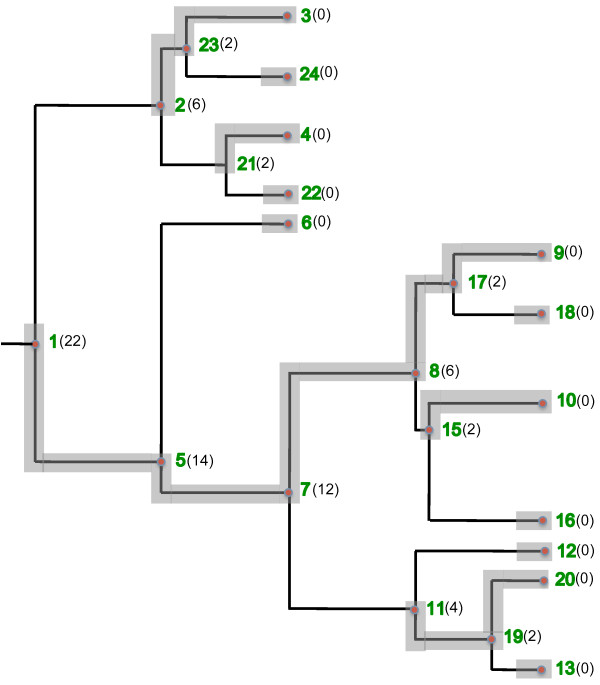
**Heavy chain decomposition.** The previously expanded tree after the heavy chain decomposition. The chains produced are: [1-5-7-8-17-9], [2-23-3], [11-19-20], [21-4], [15-10], [24], [22], [6], [18], [16], [12], [13].

For example, consider the tree in Figure 3. The value between parentheses besides the number of a node represents the number of descendants of that node (i.e. the value of *descendants*[] for that node). The cost of the chain from node 1 to node 9 is given by the following sum: 22+14+12+6+2+0 which, equals to 56. This chain is the heavy chain for node 1 since there is no other chain from node 1 to another leaf node which, has a higher cost.

Given an internal node *u*, let *children*[ *u*] be the set of child nodes of *u*. We say that a node *v*∈*children*[ *u*] is the *heaviest child* of node *u*, if for every node *x*∈*children*[ *u*], *descendants*[ *v*]≥*descendants*[ *x*] (i.e. the number of descendants of *v* is the greatest among the children of *u*). One way of obtaining the heavy chain for a node *u* is to construct a chain recursively, starting with node *u*, then inserting in the chain the heaviest child of *u*, say *v*, as the second node of the chain, then inserting the heaviest child of *v* and so forth, until a leaf node is reached. For instance, the heavy chain of node 1 in Figure 3 can be obtained by inserting node 1 in the chain. Next, inserting its heaviest child which is node 5, then inserting the heaviest child of node 5 which is node 7 and continuing this way until we reach node 9.

The heavy chain decomposition aims at partitioning the input tree into a collection of disjoint heavy chains. It is a recursive procedure that starts by obtaining the heavy chain for the root of the tree. Whenever it reaches a leaf node in a heavy chain *C* it goes back in *C*. For each node *x* in the way back in *C*, the procedure obtains the next heavy child of *x* that has not been inserted previously in any heavy chain. It then makes this heaviest child the leader of a new heavy chain and starts constructing the new heavy chain of this node. Function HeavyChainDecomposition() describes precisely the heavy chain decomposition method. If there is a tie for the heaviest child of a node, any node with the greatest number of descendants is chosen.

#### **Function**HeavyChainDecomposition(*u*)

Figure 3 shows the result of applying the function HeavyChainDecomposition() to the tree of Figure 2. Observe that the twelve heavy chains produced are highlighted and that there are heavy chains with only one leaf node. Also note that every node is associated with one and only one heavy chain.

Once the heavy chain decomposition is obtained, the LCA for any two nodes can be found efficiently as described in function LCA(). This allows us to calculate the LCA in O(log|T|) time, by skipping over chains rather than considering each branch in the chain individually.

#### **Function**LCA(*a,b*)

The whole computation of the distance matrix calculation is shown in algorithm DistanceMatrixComputation(). The algorithm starts by activating function preProcessDistance() which computes for each node *u* the distance from *u* to the root and the number of descendants of *u*. This involves traversing the tree *T* in time *O*(|*T*|). Then, the algorithm invokes function HeavyChainDecomposition(), which also executes in time *O*(|*T*|). Next, the distance matrix is finally computed. The computation of the distance matrix (lines 5 to 7 of the algorithm) involves determining the LCA for each pair of nodes. The time of this computation is *O*(|*T*|^2^) multiplied by the time necessary to obtain the LCA for each pair of nodes. We have that the worst case for function LCA() occurs when the tree is a balanced tree, that is, a tree *T* with |*T*| nodes organized in such a way that *T* has the smallest height among all possible organizations of trees with |*T*| nodes. In this case, depending on which heavy chain each node of a pair is, it is possible that function LCA() iterates through nodes along the height of the tree. Consequently, the execution time of function LCA() in the worst case corresponds to the height of the balanced tree which is O(log|T|). Thus, the total execution time of the distance matrix computation is$O(|T|)+O(|T|)+O(|T{|}^{2}\;\cdot \log|T|)$, which equals to $O(|T{|}^{2}\;\cdot \log|T|)$.

Most applications in phylogeny involve computing only the distance between species that correspond to leaf nodes in the phylogenetic tree. The distance matrix containing only leaf nodes is referred to as *patristic distance matrix*. Algorithm DistanceMatrixComputation can be easily adapted to compute patristic distance matrix. All that is needed is an additional condition in the test of line 5 of the algorithm, requiring that both *a* and *b* is leaf nodes. If we consider |*T*_*ℓ*_| as the number of leaf nodes in a phylogenetic tree *T* with |*T*| nodes then, the execution time of algorithm DistanceMatrixComputation to compute the patristic distance matrix is $O(|T_{\ell }{|}^{2}\;\cdot \log|T|)$.

#### **Algorithm** DistanceMatrixComputation(*T*)

## Results and discussion

The experiments were conducted using an AMD Phenom II x4 925 2.85 GHz, 4 GB RAM, and the Linux (Ubuntu 12.04) operating system. Our algorithms were implemented using the C++ programming language (gcc 4.6.3). We report execution times in seconds, with the reported numbers being the average of 100 independent runs. The execution times consider only processing time and do not take into account any input/output file operation. The following phylogenies were used in the experiments: Phyllostomidae (bats) [16] with 126 species, Carnivora (mammals) [17] with 209 species, Hummingbirds [18] with 304 species, and Amphibia (amphibians) [19] with 419 and 510 species (obtained by pruning the original phylogeny given in [19]).

We do not know of any other tree-expanding tool, as described in this paper, except for the AddTips R script used in [7]. Since AddTips uses APE functions [11] on top of the R environment, there is an expected overhead due to the use of an interpreted environment. For the sake of comparison, we generated a shared object that encapsulates our C++ compiled program so that it can be dynamically loaded and called from within the R environment.

Tables 1, 2 and 3 show the execution times, in seconds, when varying the number of species |*T*_*l*_|, the number *m* of trees to be expanded, and the number |*S*| of species to be inserted, respectively. The reported times were obtained with the branch-based insertion method. We noticed hardly any difference in the results produced by the node-based insertion method.

**Table 1:** **Tree generation time (seconds) for |S|=128, |T**_***ℓ***_**|=(126,…,510) and m=1,000**

**|T**_***ℓ***_**|**	**SUNPLIN-C++**	**SUNPLIN-R**	**AddTips**
126	0.32	4.25	806.80
209	0.36	4.69	1,021.49
304	0.42	5.93	1,691.40
419	0.47	7.35	2,253.90
510	0.52	7.86	2,730.90

Time in seconds for generating *m* = 1000 expanded trees, each one receiving |*S*| = 128 new species, for different input trees with |*T*_***ℓ***_| = (126, …, 510) species (Phyllostomi, Carnivora, Hummingbirds and Amphibia phylogenies).

**Table 2:** **Tree generation time (seconds) for |S|=128, |T**_***ℓ***_**|=304 and m=(1,…,10,000)**

**m**	**SUNPLIN-C++**	**SUNPLIN-R**	**AddTips**
1	0.00	0.01	1.75
10	0.02	0.06	17.44
100	0.05	0.59	174.20
1000	0.42	5.93	1,739.80
10000	4.21	59.86	17,412.06

Time in seconds for generating a varying number *m* = (1, …, 10,000) of expanded trees, given a single input tree containing |*T*_***ℓ***_| = 304 species (Hummingbirds phylogeny) and |*S*| = 128 species to be inserted.

**Table 3:** **Tree generation time (seconds) for |S|=(32,…,512), |T**_***ℓ***_**|=304 and m=1,000**

**|S|**	**SUNPLIN-C++**	**SUNPLIN-R**	**AddTips**
32	0.23	5.46	457.21
64	0.31	5.60	856.80
128	0.42	5.93	1,739.80
256	0.70	6.48	3,839.10
512	1.23	7.56	9,487.30

Time in seconds for generating *m* = 1000 expanded trees, given a single input tree containing |*T*_***ℓ***_| = 304 species (Hummingbirds phylogeny) and a varying number |*S*| = (32, …, 512) of species to be inserted.

While AddTips takes tens of minutes as the number of species to be inserted increases (Table 1), our solution takes only a few seconds in the R environment and a fraction of a second with the standalone C++ version. Inserting 128 species on a 304 species tree (Table 2) can take hours using the AddTips script, if the number of generated trees is high. On the other hand, the same task can be done in a few seconds with our C++ implementation and in less than a minute if our R version is used. As can be seen in Table 3, our implementations can handle an increase in the number of species to be inserted more smoothly than the AddTips script.

The distance matrix computation time for real data phylogenies is shown in Tables 4 and 5, for different number |*T*_*ℓ*_| of species and number *m* of trees to be expanded, respectively. We compare our implementations (C++ and R) to the APE [11] and Phylocom [13] tools. APE is a package written in R for the analysis of phylogenetics and evolution. We used its function *cophenetic.phylo* to calculate the patristic distance matrices. Phylocom is a well-known open source software (written in C) for the analysis of phylogenetic community structure and trait evolution. It calculates various metrics, including the patristic distance matrix (option *-phydist*). The execution times for APE and Phylocom were obtained by repeatedly calling their distance matrix calculation functions.

**Table 4:** **Distance computation time (seconds) for |T**_***ℓ***_**|=(126,…,510) and m=1,000**

**|T**_***ℓ***_**|**	**SUNPLIN-C++**	**SUNPLIN-R**	**APE**	**Phylocom**
126	0.55	2.05	39.92	1.33
209	1.58	3.41	90.40	4.06
304	3.85	6.97	150.90	15.89
419	7.89	15.58	211.32	36.49
510	12.11	25.31	289.15	51.69

Time in seconds for the computation of *m* = 1000 distance matrices, for different input trees with |*T*_***ℓ***_| = (126, …, 510) species (Phyllostomi, Carnivora, Hummingbirds and Amphibia phylogenies).

**Table 5:** **Distance computation time (seconds) for |T**_***ℓ***_**|=304 and m=(1,…,10,000)**

**m**	**SUNPLIN-C++**	**SUNPLIN-R**	**APE**	**Phylocom**
1	0.00	0.01	0.15	0.02
10	0.04	0.08	1.50	0.18
100	0.39	0.70	15.05	1.64
1,000	3.85	6.97	150.90	16.47
10,000	38.51	69.69	1,519.00	164.69

Time in seconds for the computation of a varying number *m* = (1, …,10,000) of distance matrices, each one associated with a |*T*_***ℓ***_| = 304 species (Hummingbirds phylogeny) expanded tree.

To calculate the distance matrices associated to 1,000 trees (Table 4), our C++ implementation is at least 20× faster than APE and 4× faster than Phylocom. When called from within the R environment our solution is still competitive, being approximately 10× faster than APE and 2× faster than Phylocom. As we increase the number of trees to be analyzed, the APE implementation can take tens of minutes (see last row of Table 5), while our C++ implementation takes less than 1 minute. Phylocom also shows a good performance but is still 4× slower than our C++ implementation and 2× slower than our R solution.

If we consider a large-scale simulation consisting of the generation of 10,000 expanded trees (last row of Table 2) and the corresponding distance matrices calculations for a 304-species tree (last row of Table 5) in the R environment, the current tools would take hours to complete, while our solution is able to get the job done in approximately 2 minutes. The total time can be further improved if the C++ code is used directly, i.e., without being called from the R environment.

We also generated random trees to study the behavior of the algorithms with increasing number of taxa. The computation time in seconds for the C++ code is shown in Tables 6 and 7 for randomly generated phylogenies. The results for expanding and calculating distance matrices of random trees with 100, 250, 500, 750 and 1,000 species are shown in Table 6. For each tree, 100 species are inserted and the resulting expanded trees are used in the distance computation algorithm. This process is repeated 1,000 times, that is, 1,000 copies are produced for each input tree. The results are similar to those using real data phylogenies (see Tables 1 and 4). For the 1,000-species tree (last row of Table 6), the generation of 1,000 expanded (1,100 species) trees takes less than a second, and when the distance matrix computation is taken into account, the overall time is less than a minute. The performance of the algorithms for a varying number of expanded trees is reported in Table 7. An input 500-species tree is expanded with 100 species and the process is repeated 1, 10, 100, 1,000 and 10,000 times. The distance computation algorithm then processes the resulting expanded trees, each one containing 600 species. The results follow the same pattern as shown in Tables 2 and 5, with the distance computation taking much longer than the expansion phase, as predicted by our complexity analysis. The generation of 10,000 expanded trees (last row of Table 7) takes less than 5 seconds, while the distance matrix computations requires no more than 2.5 minutes.

**Table 6:** Tree generation and distance matrix computation time (seconds) for SUNPLIN-C++ using different randomly generated phylogenies

	**Tree expansion**	**Distance matrix**
**|T**_***ℓ***_**|**	**for |T**_***ℓ***_**|**	**for |** ***T***_***ℓ***_**|+|S|**
100	0.25	1.98
250	0.32	6.00
500	0.44	14.45
750	0.56	28.12
1,000	0.67	44.80

Time in seconds for generating *m* = 1,000 expanded trees and to calculate distance matrices. The input trees contain |*T*_***ℓ***_| = (100, …, 1,000) species (randomly generated phylogenies) and each one is expanded with the insertion of |*S*| = 100 species.

**Table 7:** Tree generation and distance matrix computation time (seconds) for SUNPLIN-C++ using a randomly generated phylogeny

	**Tree expansion**	**Distance matrix**
**m**	**for |T**_***ℓ***_**|=500**	**for |T**_***ℓ***_**|=500+|S|**
1	0.01	0.02
10	0.02	0.15
100	0.06	1.43
1,000	0.44	14.45
10,000	4.27	144.27

Time in seconds for generating a varying number *m* = (1, …,10,000) of expanded trees and to calculate distance matrices. The input tree contains |*T*_***ℓ***_| = 500 species (randomly generated phylogeny) and is expanded with the insertion of |*S*| = 100 species.

## Conclusions

In this article, we proposed a new computational tool to conduct large-scale phylogenetic comparative simulations that take uncertainty into account. We presented efficient algorithms and implementations to generate random expanded trees and to calculate patristic distance matrices, both commonly used in large-scale statistical analyses. The algorithm proposed for generating random expanded trees performs a single tree traversal for each tree being expanded, inserting the provided phylogenetic uncertain taxa (PUT) randomly into the partially known tree. The calculation of the patristic distance matrices is performed using a heavy chain decomposition, which structures the expanded trees in a way that avoids some of the calculations along the branches of the tree. These strategies were implemented using C++ language and in R system through loadable shared objects, and compared to some of the current tools used for similar tasks. Our experimental evaluation showed that the tree expansion proposed could be done in only a few seconds, which is at least three orders of magnitude faster than the other tool analyzed. Our results also showed that the distance matrix calculation of our implementation could be up to one order of magnitude faster than the other similar tools. Given the wide adoption of the R package by the biology community, our implementations allow one to take uncertainty into account in their analyses, and seamlessly use a number of additional statistical analyses available in R. Overall, our results showed that the proposed algorithms and implementations can play an important role in helping biologists conduct their comparative phylogenetic simulations with uncertainty.

## Availability and requirements

**Project name:** SUNPLIN.**Homepage:**https://sourceforge.net/projects/sunplin/.**Webserver:**http://purl.oclc.org/NET/sunplin/. **Operating system(s):** Linux, MacOS and Windows.**Programming language:** C++.**Other requirements:** sunplin-r requires R.Windows users need also the Rtools.**License:** GNU General Public License version 2.0 (GPLv2).**Any restrictions to use by non-academics:** none.

## Abbreviations

PUT: Phylogenetic uncertain taxa; MDCC: Most derived consensus clade; PDM: Patristic distance matrix; LCA: Lowest common ancestor; DFS: Depth first search.

## Competing interests

The authors declare that they have no competing interests.

## Authors’ contributions

WSM and TFR conceived the project and contributed to the writing of the manuscript. WSM and WCC designed the algorithms. WCC implemented and carried out all computational experiments. TCR and HJL formalized the algorithms and provided feedback on the software development and manuscript. All authors read and approved the final manuscript.
